# Two-Year Accelerated Corneal Cross-Linking Outcome in Patients with Progressive Keratoconus

**DOI:** 10.1155/2015/325157

**Published:** 2015-01-06

**Authors:** Arleta Waszczykowska, Piotr Jurowski

**Affiliations:** Department of Ophthalmology and Vision Rehabilitation, Medical University of Łódź, Żeromskiego 113, 90-549 Łódź, Poland

## Abstract

*Purpose*. To evaluate the long-term results of accelerated corneal cross-linking (CXL) in patients with progressive keratoconus. *Methods*. Sixteen patients underwent accelerated CXL at 6 mW/cm^2^ for 15 minutes in one eye. The follow-up visits were scheduled on 7 days, 14 days, and 3, 12, and 24 months after the treatment. *Results*. There were no significant differences (*P* > 0.05) between preoperative and 2-year postoperative mean values, respectively, in terms of uncorrected visual acuity, best spectacle-corrected visual acuity, maximum keratometry *K*_max⁡_, minimum keratometry *K*_min⁡_, corneal astigmatism, and corneal eccentricity index. We noted a significant flattening of the cornea in 18.7% of patients with a higher preoperative *K*_max⁡_ value (>50 D) and its steepening in patients with a lower *K*_max⁡_ value (<50 D) (6.25%). There was no significant difference in the central corneal thickness and the apical corneal thickness preoperatively and 2 years postoperatively. The mean demarcation line depth was 282 ± 11 *μ*m. Persistent corneal haze was noted in 25% of patients. *Conclusions*. Accelerated CXL appears to be a relatively effective procedure for the treatment of keratoconus in 2-year follow-up.

## 1. Introduction

Cross-linking (CXL, X-linking) is a low-invasive procedure designed to strengthen the corneal structure and stop the progression of keratoconus. The method was applied for the first time at Dresden University of Technology (Germany) in 1998 [1]. The efficacy and safety of the procedure were confirmed in numerous clinical trials [2–4]. From the times of “Dresden Protocol” until now, multiple variations of the standard procedure technique have been introduced and consistent improvement is still being searched for.

In this context, the fact that the standard procedure is long-lasting and troublesome for both the patient and operating ophthalmologist is highlighted. The reduction of CXL procedure duration may be achieved through a shorter riboflavin administration time with improved corneal penetration (e.g., thanks to additional iontophoresis) or through the application of higher UV doses (accelerated cross-linking, fast cross-linking, and rapid cross-linking). The theoretical background of the latter modification is based on the photochemical reciprocity law (Bunsen-Roscoe law), according to which rate of the photochemical and photobiological reaction is directly proportional to the total dose of radiation energy. The thesis allows for the assumption that similar effect of corneal cross-linking, similar to that achieved with the standard CXL method, may be achieved with various intensity and duration of UVA irradiation, provided that an identical dose of energy is delivered [5]. Corneal biomechanical properties test results, after standard CLX procedure, in which the total UVA energy delivered to the corneal was 5.4 J/cm^2^, indicate some increase in corneal stiffness. On the other hand, experimental trials proved that exceeding the safe radiation dose (5.4 J/cm^2^) may significantly increase the risk of intraocular complications [4]. Modifications of the CXL procedure consisting in the proportional change of UVA irradiation duration and intensity, with a constant safe dose of total radiation dose energy, seem to be the proper direction.

*Aim of the Study*. The aim of the paper was the long-term assessment of the efficacy of the accelerated cross-linking procedure in patients with progressive keratoconus.

## 2. Material and Methods

The study involved 16 patients (mean age 33.00 ± 10.51 years; 15 men, 1 woman) with documented progression of keratoconus within the last 12 months (an increase in astigmatism or myopia by 1 D or an increase in *K*_max⁡_ by 1 D). Each patient underwent the accelerated cross-linking procedure in one eye. The stage of keratoconus was determined according to Amsler-Krumeich's classification. Inclusion criteria for this study were corneal thickness of at least 400 *μ*m at the keratoconus apex, 2nd or 3rd stage keratoconus according to the abovementioned classification, and the withdrawal of wearing rigid contact lenses for 4 weeks before the procedure and the entire follow-up period after the procedure (24 months). All the patients qualified for the procedure were of age and signed informed consent for the surgery.

Exclusion criteria included corneal thickness < 400 *μ*m, dry eye syndrome, history of viral corneal inflammation, past ophthalmologic surgeries, cornea guttata, corneal scars, pregnancy, autoimmune diseases, and chronic corticosteroid therapy. The study was conducted in line with the ethical principles specified in the Declaration of Helsinki of 1964. Approval of relevant Bioethics Committee was obtained (number RNN/508/11/KB).

Before the procedure and during all the follow-up visits, all the patients underwent the following: an assessment of uncorrected visual acuity (UCVA) and best spectacle-corrected visual acuity (BSCVA) for distant vision (LCD Frey CP-400, Frey Sp.J., Piaseczno, Poland), assessment of the anterior and posterior segment of the eye in a slit lamp (SL-D2 Topcon Inc., Paramus, NJ, USA) using Volk 90 D lens (Volk Optical, Mentor, OH, USA), and Placido's disk videokeratography (Keratograf 4, Oculus Inc, Wetzlar, Germany), as well as ultrasound pachymetry (handheld device PacScan 300AP, Sonomed Inc., Lake Success, NY, USA) and spectroscopic optical coherence tomography (SOCT, 3D Optical Coherence Tomograph 1000 MARK II, Topcon Inc., Paramus, NJ, USA).

Visual acuity was determined using Snellen charts. For the purpose of statistical analysis, the Snellen visual acuity was converted to the corresponding logarithm of the minimum angle of resolution (log MAR) value using standard conversion tables.

Corneal epithelium restoration time and features of corneal oedema and its transparency were assessed, among others, following the CXL procedure. The corneal haze stage was scored according to Hanna's 5-item scale (stage 0: clear cornea without any haze; 1: minimal, focal haze of stroma; 2: scattered, well-visible stromal haze; 3: diffuse stromal haze, partially obscuring iris details; 4: focal and diffuse haze obscuring iris details).

Simulated *K*_max⁡_ (Sim *K*_max⁡_), simulated *K*_min⁡_ (Sim *K*_min⁡_), astigmatism value, corneal eccentricity index, and keratoconus stage and its point apex were determined in videokeratography.

Ultrasound pachymetry was applied to measure the corneal thickness within its centre and keratoconus apex, determined by videokeratography. Measurements in each of these points were made three times and the results were averaged.

The SOCT examination was applied to determine the demarcation line depth in the corneal centre. The demarcation line depth was measured from the epithelium to the hyperreflection line within the corneal stroma during follow-up visits 12 and 24 months after the CXL procedure.

During the cross-linking procedure qualification visit, all the patients underwent Schirmer's test under anaesthesia and the invasive tear break-up time (BUT) test involving the administration of fluorescein into the conjunctival sac. Follow-up tests, including a complete ophthalmological examination, were performed 7 days, 14 days, and 3, 12, and 24 months after the procedure.

### 2.1. Mode of the Procedure

30 minutes before the procedure, the patient was given Pyralginum, an oral analgesic (metamizole sodium 500 mg, Polpharma OTC, Starogard Gdański, Poland). To narrow the pupil and provide additional protection of the lens and retina, 1 drop of 2% pilocarpine solution (Pilocarpinum, pilocarpine hydrochloride, WZF Polfa Warszawa, Poland) was instilled into the conjunctival sac. During that time, a proxymetacaine hydrochloride topical analgesic (Alcaine 5 mg/mL, Alcon Laboratories Inc., Fort Worth, TX, USA) was applied 5 times. The cross-linking procedure was performed in operating theatre conditions. Following the washing and sealing of the surgical field with sterile drape, the conjunctival sac was rinsed with 10% povidone iodine solution (Betadine, EGIS Pharmaceuticals, Budapest, Hungary). Following the mechanical corneal epithelial abrasion in the corneal centre, in the 8.0 mm diameter field, by means of single-use hockey-stick blade (Grieshaber; Alcon, Schaffhausen, Switzerland), an intraoperative corneal pachymetry (PacScan 300AP) was performed in the keratoconus apex determined by videokeratography. When the corneal thickness exceeded 400 *μ*m, a 30-minute application of 0.1% isotonic riboflavin solution (Opto Ribolink, Opto Global Pty Ltd., Adelaide, Australia), instilled into conjunctival sac every 2 minutes, was commenced. When the corneal thickness on the keratoconus apex was ≤400 *μ*m, a 0.1% hypoosmolar riboflavin solution was administered. Following the 30-minute application, the corneal pachymetry was repeated to ensure that the corneal thickness exceeded 400 *μ*m. For the period of the riboflavin application, the lid speculum was removed to allow the patient's eye to blink. Following the check-up for the presence of riboflavin in the anterior chamber under a surgical microscope (Leica M844, Leica Microsystems Inc, Allendale, NJ, USA), the cornea was irradiated with UV light at the power of 6 mW/cm^2^ and a 365 nm wavelength (UVA) (Opto XLink-Corneal Crosslinking System, Opto Electronica S/A, Sao Paulo, Brazil) for 15 minutes. During exposure to UVA radiation, an isotonic or hypoosmolar riboflavin solution was applied to the abraded epithelium field every 3 minutes. Following the procedure, 1 drop of tropicamide solution (Tropicamidum 10 mg/mL, WZF Polfa Warszawa, Poland) and levofloxacin solution (Oftaquix 0.5%; Santen Oy, Tampere, Finland) was instilled into the conjunctival sac, and a therapeutic contact lens (Acuvue Oasys, Johnson & Johnson, Limerick, Ireland) was applied.

The following medicines were applied during the postoperative period: levofloxacin (Oftaquix 0.5%) 4 times a day for 7 days, dexamethasone 1 mg/mL (dexamethasone 0.1%, WZF Polfa Warszawa, Poland) 4 times a day for 21 days, and 0.15% sodium hyaluronate (Hyabak, Thea Laboratories, Clermont-Ferrand, France) 5 times a day for 21 days and 3 times a day for the subsequent 70 days. The patients were examined by means of slit lamp on a daily basis until the restoration of corneal epithelium.

### 2.2. Statistical Analysis

The arithmetic mean and standard deviation as well as minimum and maximum values were provided for quantitative variables. The frequency of the occurrence of the analysed variable categories was calculated and provided as percentages. The differences between the average values of quantitative variables at various stages of the study were assessed by means of the analysis of variance for repeated measures (Friedman ANOVA) with a dedicated post hoc test, the Wilcoxon rank test, and Mann-Whitney* U* test (as the distribution of these variables in the Shapiro-Wilk test differs from the normal distribution). The relationship between the quantitative variables was assessed by means of Spearman's rank correlation coefficient. The significance level assumed for analyses was *α* = 0.05. The values of quantitative variables were presented as mean ± standard deviation. The statistical analysis was performed by means of statistical software (Statistica v.10, Stat Soft Inc., Tulsa, OK, USA, 2010).

## 3. Results

All the patients achieved normal results of Schirmer's test (>15 mm) and tear break-up time (BUT) test (>10 s). The mean time until complete reepithelisation was 3 days; after that period, the protective contact lens was removed. In 12 eyes (75% of the patients), the slit lamp examination on the 7th day after the procedure revealed oedema of the anterior corneal portion; the oedema was resolved within 3 months in all the patients. During the entire follow-up period, the fundus examination did not reveal any abnormalities in any of the patients.

Table 1 shows the primary preoperative and postoperative outcomes, which are summarized.

### 3.1. Visual Acuity

**Figure 1:**
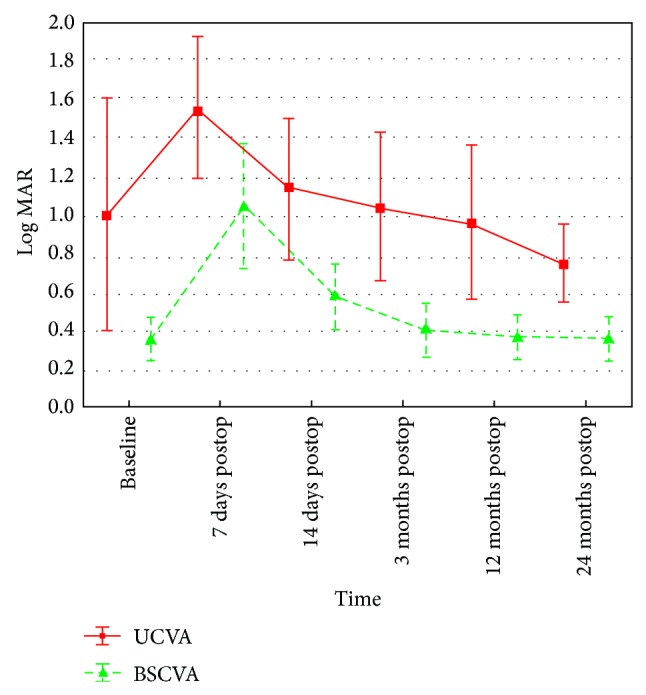
Changes in UCVA and BSCVA between baseline and 7 days, 14 days, and 3, 12, and 24 months after accelerated cross-linking in patients with progressive keratoconus.

**Figure 2:**
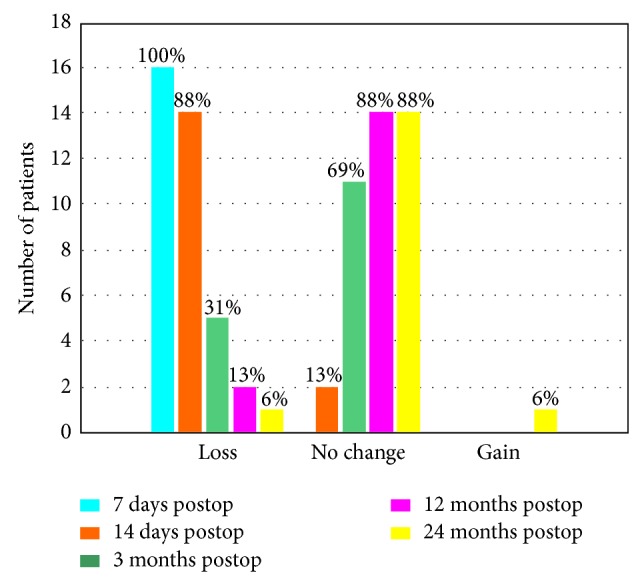
Gain/loss of BSCVA between baseline and 7 days, 14 days, and 3, 12, and 24 months after accelerated cross-linking in patients with progressive keratoconus.

The mean UCVA and BSCVA deteriorated significantly within the first 2 weeks after the procedure and did not differ significantly from the baseline in the 12th and 24th months (*P* > 0.05) (Table 1, Figure 1). During 24-month follow-up, we did not observe any case of improvement or deterioration of BSCVA by more than one Snellen chart line (Figure 2).

### 3.2. Changes in Corneal Topography

**Figure 3:**
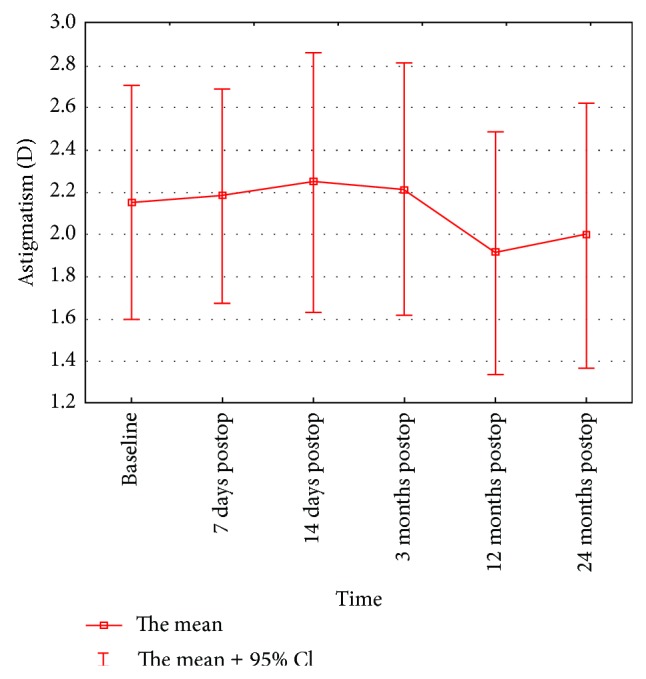
Changes in astigmatism value between baseline and 7 days, 14 days, and 3, 12, and 24 months after accelerated cross-linking in patients with progressive keratoconus.

**Figure 4:**
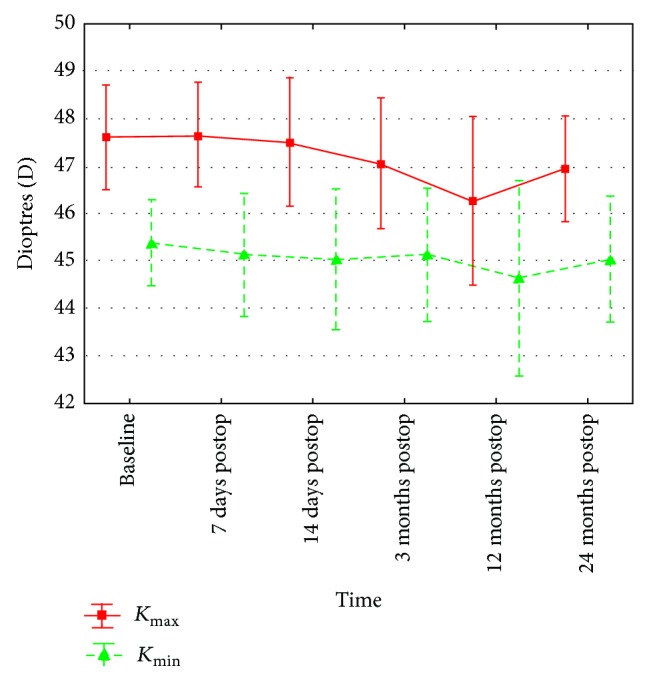
Changes in maximum and minimum keratometry value (*K*_max⁡_, *K*_min⁡_) between baseline and 7 days, 14 days, and 3, 12, and 24 months after accelerated cross-linking in patients with progressive keratoconus.

The differences in corneal astigmatism were not statistically significant at any stage of the study (*P* = 0.128) (Table 1, Figure 3). A tendency toward the reduction of the Sim *K*_max⁡_ from the baseline value to 12-month value was visible; in the 24th month, the value was similar to the baseline. Also, no significant differences in Sim *K*_min⁡_ values at particular stages of the study were found (*P* = 0.11) (Table 1, Figure 4).

Significant corneal flattening in the form of the reduction of Sim *K*_max⁡_ by >1 D was noted in 3 patients (18.7%). In 1 eye (6.25%), the difference between the baseline Sim *K*_max⁡_ and 24-month value (Δ Sim *K*_max⁡_) was 5.2 D, in another eye (6.25%) Δ Sim *K*_max⁡_ was 2 D, and in the last eye (6.25%) Δ Sim *K*_max⁡_ was 1.5 D. Significant change in the form of the reduction of Sim *K*_min⁡_ > 1 D was found in one patient only (6.25%), with Δ Sim *K*_min⁡_ equal to 5.4 D.

The mean corneal eccentricity index did not change in terms of statistical significance during the entire follow-up period (*P* = 0.3249) (Table 1).

### 3.3. Corneal Thickness Measurement

**Figure 5:**
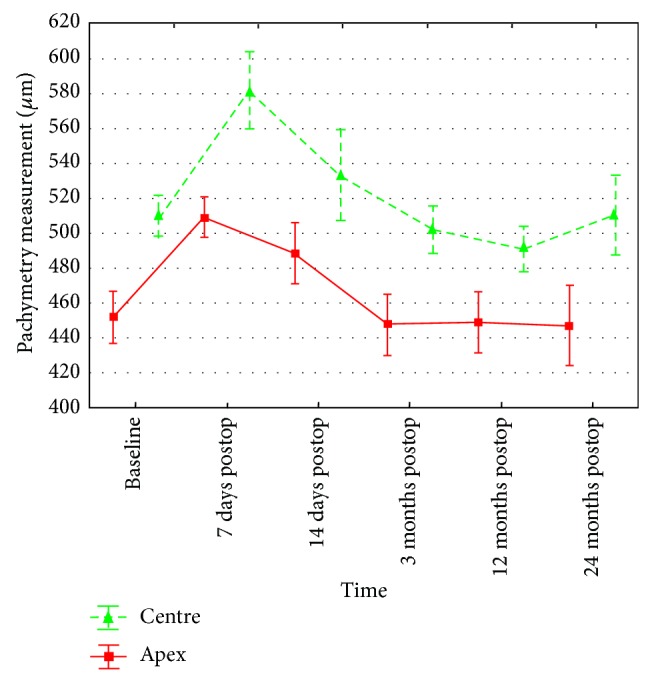
Changes in central and apex corneal thickness measurements between baseline and 7 days, 14 days, and 3, 12, and 24 months after accelerated cross-linking in patients with progressive keratoconus.

In 3 patients (18.7%), the corneal thickness at the keratoconus apex after the epithelial abrasion was lower than 400 *μ*m. Statistical analysis revealed a significant increase in the thickness in the centre and keratoconus apex within the first 7 days after the CXL procedure, but it did not reveal any differences relative to the baseline in the measurement performed 14 days and 3 months after the procedure. 12 months after the procedure, the central corneal thickness was reduced significantly and, after subsequent 12 months (24-month follow-up after the CXL), the pachymetry results returned to the baseline values. The corneal thickness at the keratoconus apex 12 and 24 months after the procedure was comparable to the baseline values (Table 1, Figure 5).

The demarcation line depth after CXL procedure was determined* during the SOCT examination*. The difference in demarcation line depth 12 months and 24 months after the procedure was not statistically significant (*P* > 0.05).

The analysis of the correlations between the demarcation line depth 24 months after the CXL procedure and the baseline corneal thickness (*r* = −0.145, *P* > 0.05), baseline keratometry: Sim *K*_max⁡_ (*r* = 0.116; *P* > 0.05) and Sim *K*_min⁡_ values (*r* = −0.113; *P* > 0.05), as well as the corneal thickness and keratometry results (corneal thickness: *r* = 0.101; *P* > 0.05; Sim *K*_max⁡_: *r* = −0.371, *P* > 0.05; Sim *K*_min⁡_: *r* = −0.521, *P* > 0.05) did not reveal any statistical significance.

There was also no correlation between the demarcation line depth and the baseline UCVA (*r* = −0.519; *P* > 0.05) and BSCVA (*r* = −0.502; *P* > 0.05) as well as the UCVA and BSCVA values 24 months after the CXL procedure (UCVA: *r* = −0.486, *P* > 0.05; BSCVA: *r* = −0.502, *P* > 0.05).

### 3.4. Other Findings

In four eyes (25% of patients), the central corneal haze (stage 1 in Hanna scale) persisted in the 2-year follow-up that did not resolve despite intensive topical therapy with steroid eye drops. The statistical analysis did not reveal any relationship between the occurrence of corneal haze after the CXL procedure and the preoperative values of the UCVA, BSCVA, corneal thickness at the keratoconus apex, Sim *K*_max⁡_, Sim *K*_min⁡_, and demarcation line depth (*P* > 0.05). No relationship between the occurrence of corneal haze and the final achieved UCVA and BSCVA was demonstrated (*P* > 0.05).

## 4. Discussion

The majority of studies evaluating the safety and efficacy of modifications of the standard CXL mode involve experimental studies on the porcine cornea. There are only several reports on the outcome of accelerated cross-linking in keratoconus patients available in medical literature [6–14]. Schumacher et al. demonstrated the equal efficacy of the accelerated cross-linking and standard CXL procedure on the porcine cornea, with UVA irradiation at a power of 10 mW/cm^2^ for 9 minutes [5]. Wernli et al. noted an increased corneal stiffness in line with the increase in UVA intensity to 45 mW/cm^2^ and a decrease in the exposure time to 2 minutes. With the application of a higher UVA intensity (above 50 mW/cm^2^) and shorter exposure time, no significant changes were found in corneal stiffness, when compared with an untreated cornea [15]. On the other hand, the most recent reports on porcine cornea studies indicate that any increase in UVA intensity and shorter exposure time, as compared to the standard procedure, diminish the effect of the cross-linking procedure [16], which is explained by increased oxygen consumption and, consequently, lower efficiency of photochemical reactions during the accelerated CXL procedure [17]. This is supported by results indicating the complete lack of effects of the CXL performed in anaerobic conditions [18]. It may be speculated that the results of experimental studies on enucleated animal eyeballs cannot be referred in full to the outcome achieved in keratoconus patients following the accelerated CXL procedure. This results from the fact that, in ex vivo studies, the cornea may be in conditions of better oxygen supply. Moreover, the quality and efficiency of photochemical reactions in the cornea of patients with ectasia may be different.

The results of our 2-year follow-up after the accelerated CXL procedure did not show significant improvement in visual acuity. The UCVA and BSCVA deteriorated significantly within the first 3 months after the procedure. The visual acuity achieved values comparable to the baseline as late as 12 months after and did not improve further until the end of the 2-year period. We also did not note statistically significant differences in pre- and postoperative astigmatism, Sim *K*_max⁡_, Sim *K*_min⁡_, and corneal eccentricity index values at any stage of the study. In line with our results, Tomita at al. did not observe significant reduction of corneal astigmatism and *K*_max⁡_ values in the 1-year follow-up after the accelerated CXL procedure (in modification involving 30 mW/cm^2^ UVA for 3 minutes). Interestingly, the *K*_max⁡_ value increased significantly 3 months after the procedure, while it later returned to baseline value [6]. Kanellopoulos obtained different results, indicating a significant improvement in visual acuity in the 4-year follow-up of 21 eyes after the accelerated CXL procedure (in modification involving 7 mW/cm^2^ UVA for 15 minutes) [7]. The mean UCVA value changed from 20/60 to 20/40 and BSCVA from 20/30 to 20/25. The mean astigmatism value decreased by 2.9 D, and the *K*_max⁡_ value was reduced from 49.5 to 46.1 D. Such a divergence of the study results may result from the fact that Kanellopoulos applied different UVA doses and epithelial removal techniques, namely, phototherapeutic keratectomy (PTK). The author suggests that the application of a higher UVA power during the procedure may provide some advantage over the standard CXL procedure due to reduced keratocyte destruction [7]. However, such a claim is not confirmed by results of the corneal confocal microscopy in patients following the accelerated CXL procedure (in modification involving 30 mW/cm^2^ UVA for 3 minutes) that revealed more intense morphological changes than following a standard CXL procedure 1 month after treatment. The differences were visible in portion of the anterior-mid corneal stroma and involved increased keratocytes apoptosis, tissue hyperreflectivity, a honeycomb-like appearance, and more numerous and darker circular lacunae. After 6 months of the follow-up in the corneas treated with the standard and accelerated CXL, similar morphological changes were observed [6, 8]. Thus, we presume that the outcome of the accelerated CXL procedure observed by Kanellopoulos was associated with the application of the PTK and, consequently, the additional removal of Bowman's membrane. It may be supposed that it allowed for the occurrence of a polymerisation reaction in deeper corneal layers.

In our study, the *K*_max⁡_ ranged from 43.3 to 51.2 D. The clinically significant decrease in *K*_max⁡_ value following the accelerated CXL procedure was only noted in 18.7% of patients. The preoperative *K*_max⁡_ values in these patients fell within the range of 50-51 D. In these cases, no postoperative corneal scars were noted that could affect the final *K*_max⁡_ values. BSCVA improvement by 1 Snellen chart line was noted in 1 patient (6.25%) only. In this case, we noted the highest reduction of *K*_max⁡_ (Δ*K*_max⁡_ = 5.9 D) and *K*_min⁡_ (Δ*K*_min⁡_ = 5.8 D) after the CXL procedure. The reduction of *K*_max⁡_ and *K*_min⁡_ values was noted 3 months after the procedure and it remained stable also in the 24-month examination. On the other hand, BSCVA improved after 24 months of the follow-up. It is particularly important that the case involved patients with the highest baseline *K*_max⁡_ value (51.2 D) among all the study subjects and 3 stages of the disease according to Amsler-Krumeich classification.

The disease progression rate following the standard CXL procedure is 7.6% and likely depends on the stage of keratoconus [19]. According to reports, keratoconus progression after the standard CXL procedure occurred in patients with a baseline *K*_max⁡_ value > 58 D [20]. In 1 patient (6.25%), disease progression was observed, involving an increase in astigmatism from 2.5 to 4.34 D together with the decrease in BSCVA by 1 Snellen chart line and decrease in corneal thickness by 12 *μ*m, with the baseline *K*_max⁡_ value of 48.7 D. Keratoconus progression occurred as early as after 1 year of follow-up, with subsequent increments of progression after 24 months of follow-up.

According to some reports, changes of corneal thickness may occur after the standard CXL procedure. In our study, the corneal thickness increased significantly within 7 days after the procedure and was comparable to the baseline value after 14 days. On the other hand, the cornea was significantly thinner in the 12th month after the procedure, while it returned to baseline values again after 24 months. The results of our study obtained 12 and 24 months after the procedure are consistent with other studies involving the standard CXL procedure [20]. The previously quoted results of the study by Kanellopoulos, involving a 4-year follow-up after the accelerated CXL procedure, showed 20% corneal thinning 1 month after the procedure, while the corneal thickness increased by 5% over baseline 18 months after the procedure [7]. Similarly, a research team from Japan demonstrated significant corneal thinning after 1 month, persisting in the 6-month follow-up, after the accelerated CXL procedure, in the modification involving 30 mW/cm^2^ UVA for 3 minutes [9]. The reasons for the corneal thickness changes after the CXL procedure should be sought in the keratocyte restoration process, the rearrangement of the corneal lamellae, anatomic and structural changes of the collagen fibres, and changes in corneal stroma (glycosaminoglycans) lasting from 12 months up to 36 months in some cases [21, 22]. Corneal ischaemia and changes in the arrangement of the new epithelium are listed among the other possible causes [23]. The results of our study concerning corneal thickness measurement may be limited by difficulties in the precise manual determination of the corneal centre and keratoconus apex. Moreover, before the measurement, 1 drop of proxymetacaine hydrochloride solution was instilled into the patient's conjunctival sac. Gao et al. reported that the application of anaesthetic drops on healthy eye surface may result in a significant false increase in corneal thickness measurement due to epithelial oedema [24]. It may be speculated that the application of anaesthetic eye drops in the early postoperative period may significantly increase the results of our measurements in the 7-day check-up after the accelerated CXL procedure. Moreover, the higher intensity of UVA radiation may damage the nerve plexus and, consequently, deteriorate the regulation of endothelial pump performance. The subbasal nerve plexus releases transported neuropeptides including substance P and calcitonin gene-related peptide. These neuropeptides may play a role in facilitating the transmission of signals to the Na/K-ATPase pumps in corneal endothelium [25]. Also, some effect of higher UVA power on endothelial cells themselves cannot be ruled out. Study results confirmed transient changes in endothelial cell density, percentages of hexagonality, and the variation coefficient of the endothelial cell area following the accelerated CXL procedure in modification involving UVA power of 18 mW/cm^2^ and the reduction of the exposure time to 5 minutes. The most significant changes were observed 7 days after the procedure, and relevant values resolved to normal range after 6 months, on average [10].

The hyperreflective demarcation line, assessed in the SOCT examination, provided for a boundary between the cross-linked cornea and its deeper layers, not affected by cross-linking. The results of our study indicate that, after the accelerated CXL procedure, the line is located at the depth of approximately 282 *μ*m within anterior corneal layers, similar to the standard CXL procedure [26]. However, reports by other authors concerning the demarcation line depth after the accelerated CXL procedure (in modification involving 30 mW/cm^2^ UVA for 3 minutes) are inconsistent. Tomita et al. noted the demarcation line at the depth of 294 *μ*m [6], while results obtained by Touboul et al. indicated its shallower location, at the depth of 100–150 *μ*m within the anterior corneal layers [8]. Kymionis et al. noted corneal stroma demarcation line depth on 288 *μ*m after accelerated CXL in modification 10 minutes with 9 mW/cm^2^ of UVA irradiation intensity and 323 *μ*m after modification procedure involving 9 mW/cm^2^ UVA for 14 minutes [13, 14]. According to literature data, hyperreflectivity of the demarcation line after the standard CXL procedure is more prominent within the first 6 months after the procedure. Later, its reflectivity becomes less prominent and more homogenous [27]. We did not find significant differences in the hyperreflectivity of demarcation line at any stage of the 24-month follow-up after the accelerated CXL procedure, which might result from the more intensive interaction of a higher UVA power on the cornea. The demarcation line is a region of transition from normal keratocytes into elongated, hyperreflective, needle-like structures and then into an area of large hyperreflective stromal bands. In 60% of eyes, these changes persist 1 year postoperatively after standard CXL [28]. We suspect that these changes may last longer after the accelerated procedure; however, it needs to be proved by confocal microscopy study.

Numerous papers addressed the occurrence of corneal haze following the standard CXL procedure. Subepithelial haze rate observed in our study (25%) is significantly higher than that observed after standard procedure that is approximately 4.5% in 6-month follow-up [27]. The incidence of corneal haze after the standard CXL procedure was observed to be positively correlated with lower UCVA and BSCVA, higher *K*_max⁡_ values, and thinner cornea in which severe corneal dehydration occurs during UVA irradiation [29, 30]. The analysis of our study results did not confirm such correlation. We observed corneal haze in patients with corneal thickness at the keratoconus apex following epithelial abrasion ranging from 409 to 421 *μ*m, who received isotonic riboflavin solution during the procedure. However, it is possible that the corneal thickness was lower in its thinnest point in these patients or the higher UVA power contributed to increased dehydration and, consequently, corneal thinning. Perhaps in the case of borderline results of corneal pachymetry, it would be more advisable to use hypoosmolar riboflavin solution during the accelerated CXL procedure. That may be supported by our finding in 3 patients with corneal thickness after epithelial abrasion lower than 400 *μ*m, who received hypoosmolar riboflavin solution during the procedure. We did not observe corneal haze in any of them.

## 5. Conclusions

In summary, the accelerated CXL procedure (in modification involving 6 mW/cm^2^ UVA for 15 minutes) seems to be relatively effective in preventing keratoconus progression after the 2-year follow-up. We did not observe any significant effect of the procedure on the improvement of functional and topographic parameters of the organ of vision. Significant corneal flattening was noted in 18.7% of patients, with higher baseline Sim *K*_max⁡_ values (>50 D). Disease progression occurred in 1 patient (6.25%) with a lower Sim *K*_max⁡_ value (<50 D). An increased complication rate, as compared to standard procedure, was noted only in the form of subepithelial corneal haze. The efficacy of the method needs to be confirmed in longer follow-up involving a larger patient group.

## Figures and Tables

**Table 1:** Clinical parameters in qualifying examination and control tests at 7 days, 14 days, and 3, 12, and 24 months after accelerated cross-linking in patients with progressive keratoconus.

	Pre-CXL (range)	7 days (range)	*P* value pre-CXL, 7 days	14 days (range)	*P* value pre-CXL, 14 days	3 months (range)	*P* value pre-CXL, 3 months	12 months (range)	*P* value pre-CXL, 12 months	24 months (range)	*P* value pre-CXL, 24 months
UCVA (log⁡MAR)	0,24 ± 0,15 (0,05–0,5)	0,07 ± 0,05 (0,004–0,2)	*P* = 0.000	0,15 ± 0,1 (0,04–0,3)	*P* = 0.004	0,19 ± 0,12 (0,06–0,4)	*P* = 0.008	0,23 ± 0,13 (0,06–0,4)	*P* > 0.05	0,24 ± 0,14 (0,05–0,5)	*P* > 0.05
BSCVA (log⁡MAR)	0,48 ± 0,2 (0,15–0,8)	0,16 ± 0,15 (0,004–0,6)	*P* = 0.000	0,33 ± 0,22 (0,08–0,8)	*P* = 0.001	0,45 ± 0,22 (0,1–0,8)	*P* = 0.043	0,47 ± 0,19 (0,125–0,8)	*P* > 0.05	0,48 ± 0,21 (0,125–0,8)	*P* > 0.05
Corneal topographic astigmatism (D)	2,16 ± 1,05 (0,9–4,5)	2,19 ± 0,96 (0,3–3,3)	*P* > 0.05	2,25 ± 1,15 (0,9–4,8)	*P* > 0.05	2,21 ± 1,12 (0,5–4,3)	*P* > 0.05	1,91 ± 1,08 (0,5–4,4)	*P* > 0.05	2,00 ± 1,04 (0,75–4,3)	*P* > 0.05
Sim *K*_max⁡_ (D)	47,61 ± 2,07 (43,3–51,2)	47,66 ± 2,06 (43,7–50,7)	*P* > 0.05	47,51 ± 2,55 (44,0–53,3)	*P* > 0.05	47,07 ± 2,41 (43,4–51,5)	*P* > 0.05	46,28 ± 3,08 (37,2–49,4)	*P* > 0.05	46,96 ± 1,93 (43,5–49,7)	*P* > 0.05
Sim *K*_min⁡_ (D)	45,39 ± 1,71 (41,7–48,2)	45,14 ± 2,44 (39,7–48,6)	*P* > 0.05	45,05 ± 2,78 (39,2–48,5)	*P* > 0.05	45,14 ± 2,44 (39,4–48,2)	*P* > 0.05	44,64 ± 3,58 (33,5–47,4)	*P* > 0.05	45,04 ± 2,3 (39,5–47,1)	*P* > 0.05
Corneal eccentricity index	0,82 ± 0,24 (0,26–1,16)	0,79 ± 0,36 (0,03–1,12)	*P* > 0.05	0,82 ± 0,31 (0,15–1,3)	*P* > 0.05	0,84 ± 0,24 (0,22–1,17)	*P* > 0.05	0,82 ± 0,29 (0,01–1,17)	*P* > 0.05	0,81 ± 0,25 (0,27–1,23)	*P* > 0.05
Pachymetry in central point (μm) in USP	509 ± 22 (476–567)	582 ± 41 (525–681)	*P* = 0.000	533 ± 49 (470–630)	*P* > 0.05	502 ± 26 (458–552)	*P* > 0.05	492 ± 23 (446–535)	*P* = 0.001	512 ± 28 (476–579)	*P* > 0.05
Pachymetry in apex point (μm) in USP	452 ± 28 (412–521)	509 ± 22 (476–567)	*P* < 0.05	489 ± 33 (435–560)	*P* > 0.05	448 ± 33 (409–537)	*P* > 0.05	450 ± 33 (409–535)	*P* > 0.05	448 ± 32 (420–530)	*P* > 0.05

BSCVA: best spectacle-corrected visual acuity; CXL: corneal collagen cross-linking; D: diopters; log⁡MAR: logarithm of the minimum angle of resolution; pre-: before treatment; Sim *K*_max⁡_: simulated keratometry in the steepest meridian; Sim *K*_min⁡_: simulated keratometry in the flattest meridian; UCVA: uncorrected visual acuity; USP: ultrasound pachymetry; data are mean ± standard deviation.

## References

[1] Raiskup F., Spoerl E. (2013). Corneal crosslinking with riboflavin and ultraviolet A. Part II. clinical indications and results. *Ocular Surface*.

[2] Spoerl E., Mrochen M., Sliney D., Trokel S., Seiler T. (2007). Safety of UVA-riboflavin cross-linking of the cornea. *Cornea*.

[3] Wollensak G., Spörl E., Reber F., Pillunat L., Funk R. (2003). Corneal endothelial cytotoxicity of riboflavin/UVA treatment in vitro. *Ophthalmic Research*.

[4] Wollensak G. (2010). Corneal collagen crosslinking: new horizons. *Expert Review of Ophthalmology*.

[5] Schumacher S., Oeftiger L., Mrochen M. (2011). Equivalence of biomechanical changes induced by rapid and standard corneal cross-linking, using riboflavin and ultraviolet radiation. *Investigative Ophthalmology and Visual Science*.

[6] Tomita M., Mita M., Huseynova T. (2014). Accelerated versus conventional corneal collagen crosslinking. *Journal of Cataract and Refractive Surgery*.

[7] Kanellopoulos A. J. (2012). Long term results of a prospective randomized bilateral eye comparison trial of higher fluence, shorter duration ultraviolet A radiation, and riboflavin collagen cross linking for progressive keratoconus. *Clinical Ophthalmology*.

[8] Touboul D., Efron N., Smadja D., Praud D., Malet F., Colin J. (2012). Corneal confocal microscopy following conventional, transepithelial, and accelerated corneal collagen cross-linking procedures for keratoconus. *Journal of Refractive Surgery*.

[9] Mita M., Waring G. O., Tomita M. (2014). High-irradiance accelerated collagen crosslinking for the treatment of keratoconus: six-month results. *Journal of Cataract & Refractive Surgery*.

[10] Cingü A. K., Sogutlu-Sari E., Çinar Y. (2014). Transient corneal endothelial changes following accelerated collagen cross-linking for the treatment of progressive keratoconus. *Cutaneous and Ocular Toxicology*.

[11] Chow V. W., Biswas S., Yu M., Wong V. W. Y., Jhanji V. (2013). Intraoperative pachymetry using spectral-domain optical coherence tomography during accelerated corneal collagen crosslinking. *BioMed Research International*.

[12] Kymionis G. D., Grentzelos M. A., Kankariya V. P. (2014). Safety of high-intensity corneal collagen crosslinking. *Journal of Cataract and Refractive Surgery*.

[13] Kymionis G. D., Tsoulnaras K. I., Grentzelos M. A. (2014). Corneal stroma demarcation line after standard and high-intensity collagen crosslinking determined with anterior segment optical coherence tomography. *Journal of Cataract and Refractive Surgery*.

[14] Kymionis G. D., Tsoulnaras K. I., Grentzelos M. A. (2014). Evaluation of corneal stromal demarcation line depth following standard and a modified-accelerated collagen cross-linking protocol. *The American Journal of Ophthalmology*.

[15] Wernli J., Schumacher S., Spoerl E., Mrochen M. (2013). The efficacy of corneal cross-linking shows a sudden decrease with very high intensity UV light and short treatment time. *Investigative Ophthalmology and Visual Science*.

[16] Hammer A., Richoz O., Mosquera S. A., Tabibian D., Hoogewoud F., Hafezi F. (2014). Corneal biomechanical properties at different corneal cross-linking (CXL) irradiances. *Investigative Ophthalmology & Visual Science*.

[17] Mrochen M. (2013). Current status of accelerated corneal cross-linking. *Indian Journal of Ophthalmology*.

[18] Kamaev P., Friedman M. D., Sherr E., Muller D. (2012). Photochemical kinetics of corneal cross-linking with riboflavin. *Investigative Ophthalmology and Visual Science*.

[19] Koller T., Mrochen M., Seiler T. (2009). Complication and failure rates after corneal crosslinking. *Journal of Cataract and Refractive Surgery*.

[20] Vinciguerra P., Albè E., Trazza S., Seiler T., Epstein D. (2009). Intraoperative and postoperative effects of corneal collagen cross-linking on progressive keratoconus. *Archives of Ophthalmology*.

[21] Mazzotta C., Traversi C., Baiocchi S. (2008). Corneal healing after riboflavin ultraviolet-A collagen cross-linking determined by confocal laser scanning microscopy *in vivo*: early and late modifications. *The American Journal of Ophthalmology*.

[22] Croxatto J. O., Tytiun A. E., Argento C. J. (2010). Sequential in vivo confocal microscopy study of corneal wound healing after cross-linking in patients with keratoconus. *Journal of Refractive Surgery*.

[23] Holopainen J. M., Krootila K. (2011). Transient corneal thinning in eyes undergoing corneal cross-linking. *The American Journal of Ophthalmology*.

[24] Gao L., Wang Z.-H., Fan H.-J., Tan L.-X. (2005). Corneal thickness is increased after topical anesthesia in myopia. *International Journal of Ophthalmology*.

[25] Arita R., Arita M., Kawai M., Mashima Y., Yamada M. (2005). Evaluation of corneal endothelial pump function with a cold stress test. *Cornea*.

[26] Yam J. C. S., Chan C. W. N., Cheng A. C. K. (2012). Corneal collagen cross-linking demarcation line depth assessed by Visante OCT after CXL for keratoconus and corneal ectasia. *Journal of Refractive Surgery*.

[27] Caporossi A., Mazzotta C., Baiocchi S., Caporossi T. (2010). Long-term results of riboflavin ultraviolet a corneal collagen cross-linking for keratoconus in Italy: the siena eye cross study. *The American Journal of Ophthalmology*.

[28] Jordan C., Patel D. V., Abeysekera N., McGhee C. N. J. (2014). In vivo confocal microscopy analyses of corneal microstructural changes in a prospective study of collagen cross-linking in keratoconus. *Ophthalmology*.

[29] Greenstein S. A., Shah V. P., Fry K. L., Hersh P. S. (2011). Corneal thickness changes after corneal collagen crosslinking for keratoconus and corneal ectasia: one-year results. *Journal of Cataract and Refractive Surgery*.

[30] Raiskup F., Spoerl E. (2011). Corneal cross-linking with hypo-osmolar riboflavin solution in thin keratoconic corneas. *American Journal of Ophthalmology*.

